# The Identification and Expression Analysis of the *Nitraria sibirica Pall*. Auxin-Response Factor (ARF) Gene Family

**DOI:** 10.3390/ijms231911122

**Published:** 2022-09-22

**Authors:** Yuxin Liu, Jingbo Zhang, Xinle Li, Liming Zhu, Ziming Lian, Hao Fang, Lu Lu, Ye Lu, Jisen Shi, Jinhui Chen, Zhaodong Hao, Tielong Cheng

**Affiliations:** 1 Key Laboratory of Forest Genetics & Biotechnology of Ministry of Education of China, Co-Innovation Center for Sustainable Forestry in Southern China, Nanjing Forestry University, Nanjing 210037, China; 2 College of Biology and the Environment, Nanjing Forestry University, Nanjing 210037, China; 3 Experimental Center of Desert Forestry, Chinese Academy of Forestry, Dengkou 015200, China

**Keywords:** auxin-response factors, drought stress, gene expression, *Nitraria sibirica*

## Abstract

*Nitraria sibirica* is a shrub that can survive in extreme drought environments. The auxin-response factors (ARFs) are a class of transcription factors that are widely involved in plant growth and development, as well as in the regulation of stress resistance. However, the genome-wide identification of the *ARF* gene family and its responses to environmental stresses, especially drought stress, in *N. sibirica* has not yet been reported. Here, we identified a total of 12 *ARF* genes in the genome of *N. sibirica*, which were distributed over 10 chromosomes and divided into three clades. Intragenome synteny analysis revealed one collinear gene pair in the *ARF* gene family, i.e., *NsARF9a* and *NsARF9b*. Cis-acting element analysis showed that multiple hormones and stress-responsive cis-acting elements were found in the promoters of *NsARFs*, suggesting that *NsARFs* may be involved in multiple biological processes. Quantitative real-time PCR (qRT-PCR) showed that many *NsARFs* had tissue-specific expression patterns, with the highest expression of *NsARF16* in the seedlings of *N. sibirica*. In addition, most of the *NsARFs* that were upregulated under drought were independent of endogenous ABA biosynthesis, whereas the response of *NsARF5* and *NsARF7a* to drought was disrupted by the ABA-biosynthesis inhibitor fluridone. These studies provide a basis for further research into how *NsARFs* in *N. sibirica* respond to hormonal signaling and environmental stresses.

## 1. Introduction

Auxin is essential for controlling plant growth and development, as it has been shown to control the processes of cell differentiation, elongation, and division in conjunction with other plant-growth regulators [1]. There are several important auxin-response genes, such as the early responsive auxin-response factor family (*ARFs*), that play a major role in the regulation of a plants’ auxin responses [2]. The structure of ARF proteins is typically composed of an N-terminal DNA-binding domain (DBD), a variable middle region (MR), and a C-terminal dimerization domain (CTD) [3]. The *ARF* DNA-binding domain may bind to the ‘TGTCTC’ motif, i.e., auxin-response elements (*AuxREs*), which are often embedded in the promoters of auxin-response genes, to transcriptionally regulate the expression of these genes that are widely involved in plant growth and development [4,5]. It has been shown in a variety of plants that varying the MR amino acid content can affect the function of ARF proteins either to promote or repress the expression of auxin-response genes. In *Arabidopsis*, ARF genes rich in glutamine and serine, proline, and glycine are shown to be suppressors or promoters, respectively [3,6]. The *ARF* DNA-binding domain may bind to TGTCTC auxin-response elements (*AuxREs*) during plant development; these elements are often associated with genes involved in growth-hormone signaling and regulate their expression [4,5]. Depending on the expression of auxin in plants, the ARF protein responds in various ways. *ARF*-regulated transcriptional activity can be inhibited by Aux/IAA protein dimers with *ARF* transcription factors in the presence of low auxin concentrations; however, the extent of the expression suppression is rather modest [7,8]. The 26S proteasome frees interacting ARF proteins from the inhibitory dimeric structure when auxin concentrations are high [9,10,11].

Studying the roles of each member of their family is crucial due to the significant role *ARFs* play in the control of plant hormone signaling. The *ARF* gene family has been identified in many plants, and the functional investigation of these members has advanced. In *Arabidopsis*, 23 members of the *ARF* family have been identified, with *AtARF7* and *AtARF9* having a role as activators of lateral root growth [12]; *AtARF1*, *AtARF2*, *AtARF3*, *AtARF4*, and *AtARF9* have been revealed to be auxin repressors, whereas *AtARF5*, *AtARF6*, *AtARF7*, and AtARF8 have been shown to be auxin-promoting factors in carrot protoplasts [3]. Furthermore, ABA’s function in *Arabidopsis* seed germination is largely dependent on the TIR1/AFB–AUX/IAA–ARF-mediated auxin signaling pathway [13]; for example, *AtARF2* is a negative regulator in the pathway of ABA regulating seed germination and primary root growth [14]. In other plant species, *ARF*’s function has been studied as well: *rice* has 25 members, of which *OsARF12* is connected to phosphate-induced auxin responses [15]; *Longan* contains 17 members, with the gene *DIARF7* being unique to leaves [16,17]. In *Cicer*, it has been found that the expression of *CaARF20* is upregulated under drought conditions [6]. Thus, *ARF* gene function has been studied in some plant species, but it is yet to be discovered in *Nitraria sibirica*. Our study is an advancement in the study of growth-hormone-regulation mechanisms in drought-resistant plants. Moreover, this will help researchers to better understand the role of *ARFs* under drought stress.

*N. sibirica* is a typical drought-tolerant plant that belongs to the genus *Nitraria Linn.*, which resides in the *Sapindales* family. Although great progress has been achieved regarding *N. sibirica* cultivation methods, physiological measurements, and resistance-regulation mechanisms, the lack of a reference gene hinders the genome-wide identification of gene families and functional genomics studies.

Abscisic acid (ABA) is a crucial endogenous plant hormone that controls plant development, stress tolerance, and stress induction (drought, high salt, and low temperature) [18]. The function of ABA in drought-resistance activities in *wheat* [19], *maize* [20,21], *soybeans* [22], and other plants has been extensively studied. Here, we identified the *ARF* gene family of *N. sibirica* and characterized the relationships between drought, ABA signaling, and *NsARF* responses.

## 2. Results

### 2.1. Genome-Wide Identification of NsARFs in the N. Sibirica Genome

To identify *N. sibirica ARF* genes, we used HMMER and the *ARF* gene structural domains as input: the Auxin Resp domain (Pfam 06507), the PB1 domain (Pfam 02309), and the B3 DNA-binding domain (Pfam 02362) were used to search the *N. sibirica* genome. We then used the SMART database and CDD search tools to determine the NsARF protein sequences manually.

We identified a total of 12 *NsARFs* in the *N. sibirica* genome. We named each member according to its *Arabidopsis* homolog (Table 1). Basic details including the chromosomal location, amino acid counts, and physicochemical characteristics of the proteins were used to identify homologs. The lengths of the NsARF protein sequences varied from 1860 aa (NsARF9b) to 3153 aa (NsARF7b); their molecular weights (MWs), from 619 kDa to 1050 kDa; their pI ranged from 5.51 to 6.71, with an average of 5.96. NsARF homologs are expressed in similar subcellular structures, as seen by the limited difference in pI value fluctuation [23].

### 2.2. Phylogenetic Analysis of NsARFs

**Figure 1:**
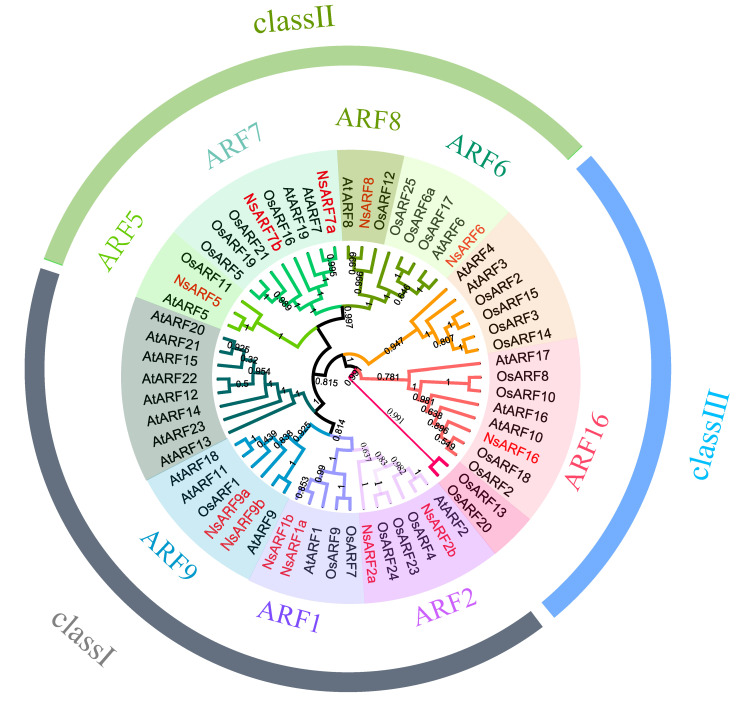
Phylogenetic relationships of the ARF family genes from *Nitraria sibirica* (*Arabidopsis thaliana*, Rice).

To investigate the phylogenetic relationship between the *NsARF* proteins and their homologs from other species, we created phylogenetic trees including the sequences of 24 *AtARFs* from *Arabidopsis*, 25 *OsARFs* from *rice*, and 12 *NsARFs* from *N. sibirica*, using the neighbor-joining (NJ) method. All the *ARFs* analyzed here were divided into three clades, i.e., I, II, and III. Class I includes six *NsARFs*, Class II includes five *NsARFs*, and the single *NsARF16* constitutes Class III (Figure 1).

### 2.3. Chromosome Distribution and Synteny Analysis of the NsARF Family

**Figure 2:**
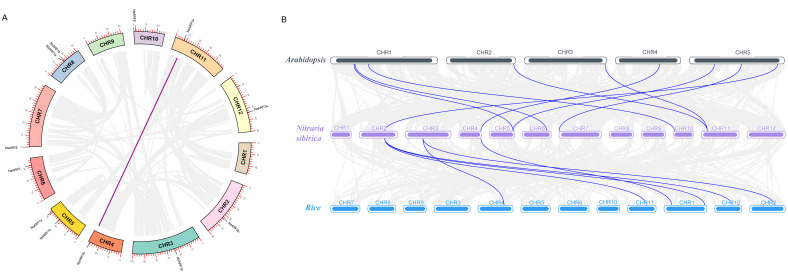
(**A**): Chromosome location and interchromosomal relationships of ARFs in *Nitraria sibirica*. (**B**): Synteny analyses between the ARFs of *Nitraria sibirica*, *Arabidopsis*, and rice.

*NsARFs* are dispersed across 10 out of its 12 chromosomes, with two on CHR5 (*NsARF7a* and *NsARF7b*), two on CHR8 (*NsARF1a* and *NsARF1b*), and one on each of CHR2, CHR3, CHR4, CHR6, CHR7, CHR10, CHR11, and CHR12 (Figure 2A). Intragenome synteny analysis showed that *NsARF9a* and *NsARF9b* are a pair of homologs. From an interspecific covariance analysis between *N. sibirica*, *Arabidopsis*, and *rice*, we identified that two groups, one of seven and one of three *NsARF* genes, were homologous to nine *AtARFs* and six *OsARFs*, respectively (Figure 2B).

### 2.4. Analysis of the NsARF Family Gene Structure

By the domain prediction of proteins encoded by *NsARFs*, we found that 10 of them contain the classic *ARF* structural domain (DBD-MR-CTD) (Table 2). Eight *NsARFs* harbor a glutamine (Q) and leucine (L)-rich middle region, implying that these proteins may be transcriptional activators.

**Figure 3:**
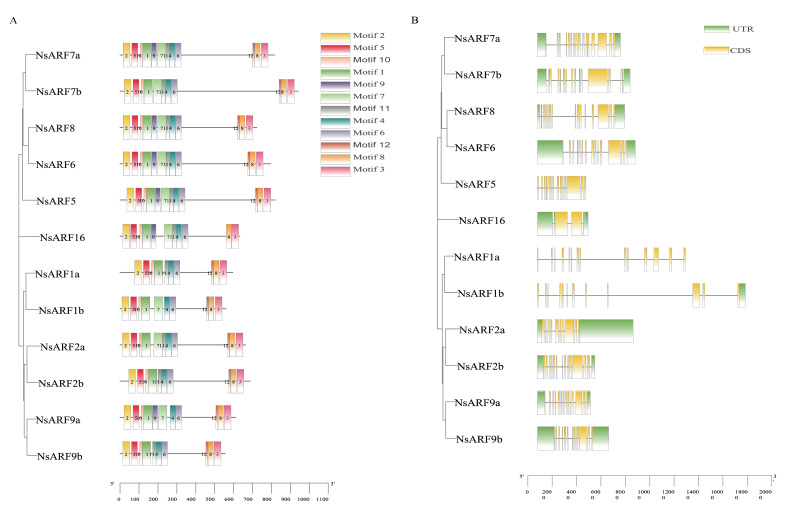
(**A**): Conservative motif distribution of *NsARF* genes. (**B**): Domain distribution of *NsARF* genes.

Motif analysis showed that 12 motifs were discovered across the NsARF protein sequences (Figure 3A and Supplemental Figure S1). There are eight motifs conserved across all the *NsARFs*, while motifs 7, 10, 11, and 12 are not identified in some sequences, of which at least six sequences contain motif 9.

Next, we analyzed the coding DNA sequence (CDS) information and untranslated regions (UTRs) of *NsARFs*. *NsARF1b* only has a 3′ UTR, and *NsARF1a* and *NsARF5* have no UTRs, while the remaining nine members have both 5′ and 3′ UTRs. The *ARF1* subclade is the only one of which its members do not have a 5′ UTR. The number of CDSs in each member sequence ranges from 3 to 16. The earlier diverging *NsARF16* gene only has 3 CDSs, whereas *NsARF7a* contains 17 CDSs (Figure 3B).

### 2.5. Prediction of Cis-Acting Elements in NsARF Family Promoter Sequences

**Figure 4:**
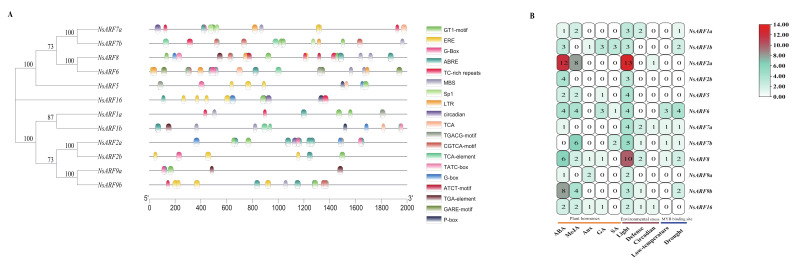
(**A**): Cis-acting element analysis of *ARF* gene in *Nitraria sibirica*. (**B**): Summary of cis-acting elements’ number and function of *ARF* gene in *Nitraria sibirica*.

We performed analyses to identify cis-acting elements in the *NsARF* gene promoters. We aimed to identify cis-acting elements of three overarching types: those related to plant hormone signaling, those involved in environmental stress responses, and MYB-binding sites (Figure 4). All the *NsARFs* contain plant hormone response elements, while seven members have MYB-binding sites linked to drought stress and eleven members have elements linked to responses to light, defense, circadian phenomena, and low temperature. The presence of light-responsive elements in 11 *NsARF* promoters and abscisic acid-responsive elements in 10 NsARF promoters suggests that the *NsARF* family is intimately linked to the abscisic acid hormone regulatory network (Table 3). In addition, seven *NsARF* promoters had the cis-element, which can bind MYBs in response to drought stress, suggesting that they might play a role in drought responses.

### 2.6. Tissue-Specific Expression Analysis of the NsARF Family

**Figure 5:**
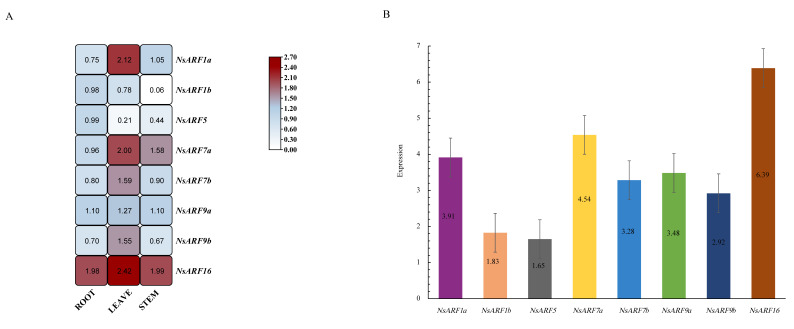
(**A**): Expression analysis of *NsARF* genes in different tissues of *Nitraria sibirica*. (**B**): Expression analysis of *NsARF* genes in one total plant of *Nitraria sibirica*.

To quantify the expression of *NsARFs* in various tissues, we chose eight *NsARFs*—*NsARF1a, 1b, 5, 7a, 7b, 9a*, and *16*—and designed customized qRT-PCR primers for each (Table 4). By performing qRT-PCR experiments, we found that *NsARFs* are widely expressed in all tissues, with high expression in the root and leaf (Figure 5A). The expression of *NsARF16* is the highest in all tissues. *NsARF5* shows considerable expression in the root, while *NsARF5* is little expressed in the leaf. *NsARF1a, 7b, 9b*, and *16* were significantly differentially expressed in the leaf compared with the other tested tissues. We summarized and determined the relative expression levels of the detected genes in the whole plant, and found that *NsARF16* showed the highest expression and *NsARF5* the lowest (Figure 5B).

### 2.7. Expression Analysis of the NsARF Family under Abiotic Stress

**Figure 6:**
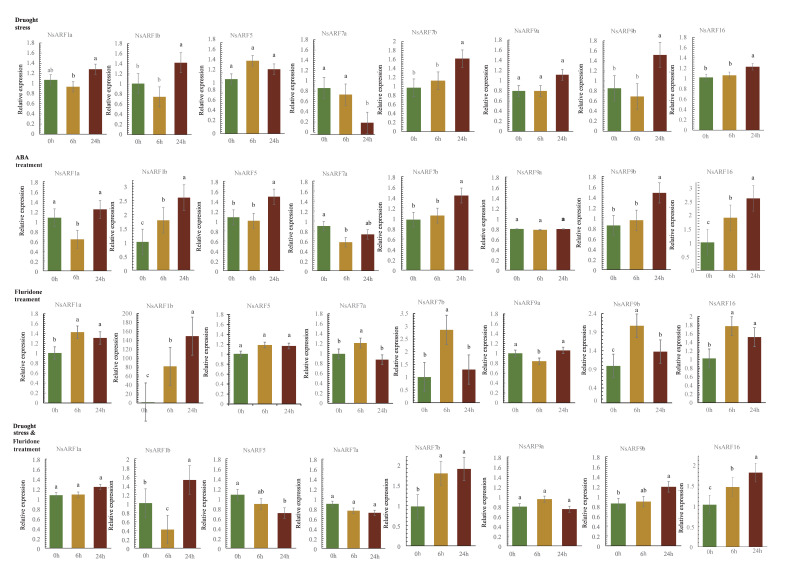
Expression profiles of *NsARFs* under abiotic stress. Values with the different letter (a–c) were significantly different when assessed using Duncan’s multiple range test (*p* < 0.05).

*N. sibirica* demonstrates a high level of drought resilience. In order to investigate the expression pattern of *NsARFs* under drought stress, we treated the seedlings of *N. sibirica* with 20% PEG 8000 to simulate conditions of drought. Under drought stress, the expression of *NsARF1b*, *NsARF7b*, *NsARF9b*, and *NsARF16* was differentially upregulated, while *NsARF7a* was the only *ARF* gene to decrease in expression. The expression levels of *NsARF1a*, *NsARF5*, and *NsARF9a* showed no significant difference (Figure 6).

ABA is a phytohormone involved in plant stress responses [18]. In order to understand how ABA influences the expression of *NsARFs*, we designed three sets of treatments. To *N. sibirica* seedlings in group A, ABA hormone was directly applied; fluridone, an ABA inhibitor, was administered to group B, and fluridone was also administered to group C, in which the plants were also undergoing drought stress (Figure 6).

The expression patterns of *NsARF1a*, *NsARF1b*, *NsARF7a*, *NsARF7b*, *NsARF9a*, *NsARF9b*, and *NsARF16* under ABA treatment are very similar to those under drought stress, with only *NsARF5* changing from no significant difference in expression upon drought stress to a significant increase upon ABA treatment. Under fluridone treatment, the expression patterns of *NsARF7a* and *NsARF7b* changed significantly compared to those under drought conditions and ABA treatment; the trend for *NsARF5* became a downward trend with respect to ABA treatment. Interestingly, the expression of *NsARF1b* was very high, but the overall trend was not different from that for the other groups. In the presence of both drought and fluridone, the expression dynamics of *NsARF1a*, *NsARF7b*, *NsARF9a*, *NsARF9b*, and *NsARF16* remained unchanged compared to those under drought conditions and ABA treatment. After the application of fluridone, the expression trends of *NsARF5* and *NsARF7a* changed significantly compared to those under only drought conditions. This finding suggests that the response of *NsARF5* and *NsARF7a* to drought was disrupted by the ABA-biosynthesis inhibitor fluridone.

## 3. Discussion

The *ARF* gene family regulates growth, hormone responses, and abiotic stress in plants [24,25], making it a critical gene family to study in order to understand plant biology. *N. sibirica* is a typical desert plant, tolerant of high drought. This is the first investigation into the *ARF* gene family in *N. sibirica*.

In this study, we used bioinformatics tools to analyze the unpublished genome of *Nitraria sibirica* and discovered 12 *NsARF* genes across 10 chromosomes, which were divided into three phylogenetic clades. Differences in the amino acid content of the MR region affect whether the gene acts as a promoter or a repressor of binding to the *AuxRE* sequence in the promoters of growth-hormone-regulatory genes [3]. According to the *NsARF* gene structure analysis, eight proteins may be transcriptional activators. The specific functional validation of these putative transcriptional activators has not yet been established. *NsARF* members are expressed in close subcellular structures, as shown by the little difference in pI value fluctuation; this will be further investigated by subcellular localization in the future.

The number of motifs in the *NsARF* gene sequences ranged from 8 to 12, indicating that the *NsARFs* are highly conserved. *NsARF16*, which is an early-diverging member within the *ARF* phylogenetic tree, has 3 CDSs, whereas *NsARF7a* has 17 CDSs. The interspecific examination of covariance revealed that eight *NsARFs* were homologous in *Arabidopsis* or *rice*. The coding region of the gene has remained stable and preserved as a result of replicative evolution that the *ARF* gene family may have undergone during speciation. By examining their cis-acting elements, it was discovered that every member of the *NsARFs* contained hormone-responsive elements. Up to 10 of these members contain ABA-response elements, and 7 *NsARFs* are predicted to bind with MYBs in response to drought stress, suggesting that they might play a role in drought response.

The function of unknown genes in a species is frequently predicted using the tissue-specific expression of genes. Therefore, the roots, stems, and leaves of *N. sibirica* were examined using qRT-RCR technology to test the *NsARF* expression levels. The results show that eight homologs have high levels of expression in the leaf and root, and low expression in the stems. Fluorescence quantitative PCR showed that *NsARF1a*, *7b*, *9b*, and *16* had tissue-specific expression patterns, with the highest expression of *NsARF16* in the total seedlings of *N. sibirica*. In the root, *NsARF5* shows significantly increased expression, while *AtARF5* is similar to *NsARF5* in the phylogenetic tree, and it has been confirmed in *Arabidopsis* that *AtARF5* is very important in the formation of the radicle [26]. It is suspected that *NsARF5* is crucial in the control of the root growth and development in *N. sibirica*. *NsARF1a*, *7a*, *7b*, *9b*, and *16* were highly expressed in the leaf. *AtARF7* has been reported to play a role in regulating leaf expansion [26], indicating that *NsARF7a* and *NsARF7b* may play roles in leaf development. Significantly, auxin responses in plants have very strong cell-type specificity, and the expression of the *ARF* gene is a part of the transduction of auxin signaling [27]. Many studies have confirmed that distinct tissues and cell types have different responses to auxin, and the specific responses of tissues or cells play an important role in hormone-mediated plant growth and development [28,29]. For example, the epidermal cell’s specific responses to auxin in the root and stem are key to those aspects of development [30,31]. At present, there is no relevant research about cell-type-specific auxin responses in *N. sibirica*. In the future, these may be revealed by the transcriptomic analysis of the responses to auxin within different tissues. On this basis, we can further explore the role of *NsARFs* in the growth and development of *N. sibirica*.

Plants under drought stress will express ABA-synthesis-related genes, which will result in ABA production mainly from root and leaf tissue [32]. The ability to synthesize is significantly higher in leaves than in roots, and ABA from the root can be transferred to the shoot. Fluridone, an inhibitor of ABA synthesis, has been used in many plants to inhibit the accumulation of ABA [33]. Deducting from the cis-acting elements that we identified in *NsARF* promoters, *NsARFs* may be intimately linked with the abscisic acid hormone regulatory network. To explore whether ABA is involved in the response of *NsARFs* to drought stress, we examined the expression changes of *NsARFs* under four sets of treatment, i.e., 20% PEG, ABA, the ABA inhibitor fluridone, and 20% PEG in combination with fluridone. In seedlings treated with ABA and subjected to drought, the ABA content increased, with a generally consistent pattern for the majority of *NsARFs*. Under fluridone treatment, the synthesis of ABA was inhibited. Interestingly, the expression of some members increased in a short time (6 h), and the expression of *NsARF1b* changed greatly, but the regulation mechanism is not clear. These phenomena are worth further exploring. The change trend of the expression of *NsARF5* was opposite to that of ABA treatment, and the decreasing trend of the expression of *NsARF7a* under drought conditions and ABA treatment was suppressed under treatment with fluridone. Different drought-related evaluation system pathways were activated in plants during drought stress and fluridone treatment; however, ABA synthesis was blocked. *NsARF1a* expression began to vary at 6 h and tended to decline in the presence of increasing ABA content and vice versa, without noticeably different results. This suggests that an early buildup of ABA has an inhibitory influence on *NsARF1a* expression. The rising trend of *NsARF1b* under the treatment of directly applied ABA or fluridone appeared at 6 h and further increased at 24 h. The expression of *NsARF1b* under drought stress combined with fluridone treatment clearly first decreased and then increased again after 24 h. It is likely that other relevant response factors in the drought regulatory pathway also significantly influence the expression of *NsARF1b*. In contrast to that under drought and ABA treatment, the expression of *NsARF5* showed a declining trend after the administration of fluridone and the decrease in *NsARF7a* expression was absent, suggesting that the expression of *NsARF5* and *NsARF7a* is linked with ABA levels. Under both drought and ABA treatment, *NsARF7b* had the same trend, and in just 24 h, the expression greatly increased. Fluridone treatment caused the expression of *NsARF7b* to considerably increase in 6 h, and it was higher than in the other two treatment groups without fluridone. ABA may suppress the expression of *NsARF7b*, which was not controlled directly by the ABA-response pathway. *NsARF9a*, *NsARF9b*, and *NsARF16* did not exhibit any appreciable differences in expression between treatments. In addition, most of the *NsARFs* that were upregulated under drought were independent of endogenous ABA biosynthesis, whereas the response of *NsARF5* and *NsARF7a* to drought was disrupted by the ABA-biosynthesis inhibitor fluridone. The auxin responses in plants were very cell-type specific and regulated epigenetically.

## 4. Materials and Methods

### 4.1. Plant Materials and Abiotic Stress Treatment

The seeds used for this investigation originated from the Inner Mongolia municipality, Dengkou County, China. For more than 60 days, the seeds were vernalized at 4 °C, covered in moist sand. The seeds were germinated at 23 °C and grew for over a month in a 4:1 soil combination of nutritious soil and perlite, until they reached a height of around 20 cm. Healthy *N. sibirica* seedlings were separated into root, stem, and leaf sections in order to gather plant components for tissue-specific analysis. Following that, plant tissues were gathered, frozen in liquid nitrogen, and kept at −80 °C. Then, the seedlings were treated at the same developmental stage with 20% PEG to simulate drought stress, 50 mg/L ABA, 50 µM fluridone, and 20% PEG with 50 µM fluridone [34,35]. We collected plant tissues after 0, 6, and 24 h. All the tissues were immediately frozen in liquid nitrogen and stored at −80 °C until RNA extraction. The dates of sample collection were 20 May to 30 May.

### 4.2. Identification of ARF Genes in N. sibirica

The Pfam codes of 3 structural domains—the Auxin Resp domain (Pfam 06507), the PB1 domain (Pfam 02309), and the B3 DNA-binding domain (Pfam 02362)—were used to extract a total of 23 sequences of *AtARFs* from the *Arabidopsis* genome (TAIR.10) [16]. According to the China Rice Date Center, 25 *OsARFs* were recovered from the *rice* genome (China Rice Date Center).

Using the HMMER software (v3.0), we searched *NsARFs* based on the genome of *N. sibirica* (unpublished). Then, the blastp software (v2.90), SMART (http://smart.embl-heidelberg.de/, accessed on 1 May 2022), and the CDD search tool (https://www.ncbi.nlm.nih.gov/Structure/bwrpsb/bwrpsb.cgi, accessed on 1 May 2022) were used to reconfirm the sequence correctness. Naming methods were adopted to identify homology with *AtARFs*. The protein’s pI and MWs (molecular weights) were discovered using a UniProt (https://www.uniprot.org/, accessed on 1 May 2022) search query.

### 4.3. Phylogenetic Analysis of ARF Proteins in N. sibirica

MEGA v10.1.8 (Temple, Philadelphia, PA, USA) was used to examine *ARFs* from *N. sibirica*, *rice*, and *Arabidopsis* to determine their phylogenetic relationship. We used MUSCLE implemented in MEGA to align the amino acid sequences and neighbor-joining algorithm to create phylogenetic trees with a bootstrap value of 1000. DANMAN v9.0 (Lynnon Corporation, San Ramon, CA, USA) was used for the multi-fragment alignment of amino acid sequences.

### 4.4. Gene Structure, Cis-Acting Element Prediction, and Chromosomal Localization Analysis of NsARFs

The MEME software (https://meme-suite.org/meme/tools/meme, accessed on 1 May 2022) was used for conserved motif structure analysis, while the Pfam sequence search algorithm (http://pfam.xfam.org/, accessed on 1 May 2022) was used to identify the location information of the protein kinase domain and NAF/FLSL domain of candidate genes. The GSDS (http://gsds.cbi.pku.edu.cn/, accessed on 1 May 2022) online analysis tool was used to analyze the structural domains of candidate genes in order to determine the chromosomal position of *NsARFs* and to visualize the findings of previous research. Cis-acting elements within the *NsARF* gene promoters were analyzed using the Plant CARE online tool (http://bioinformatics.psb.ugent.be/webtools/plantcare/html/, accessed on 1 May 2022). TBtools [36] was used to synthesize and visualize these results.

### 4.5. Expression Analysis of ARF Genes in N. sibirica

RNA was extracted using an RNA-extraction kit (Promega, Shanghai, China); the RNA was then reverse transcribed into single-stranded cDNA using a reverse-transcription kit (Vazyme, Nanjing, China). Quantitative real-time PCR (qRT-PCR) was performed using a LightCycler 480II (Roche, Basel, Switzerland) with the AceQ qPCR SYBR Green Master Mix (Vazyme, Nanjing, China). Specific qRT-PCR primers were designed in NCBI (http://bioinformatics.psb.ugent.be/webtools/plantcare/html/, accessed on 1 May 2022) to detect expression.

## Figures and Tables

**Table 1:** Summary of *N. sbirica ARF* gene family members.

Gene Name	Locus ID	Locus	Protein Length (aa)	MW (kDa)	pI	Homolog in Arabidopsis
*NsARF1a*	*NISI08G0534.1*	Chr8	1995	665	5.81	*AtARF1*
*NsARF1b*	*NISI08G0538.1*	Chr8	1875	624	6.17	*AtARF1*
*NsARF2a*	*NISI12G1517.1*	Chr12	2220	739	5.87	*AtARF2*
*NsARF2b*	*NISI02G2077.1*	Chr2	2298	765	5.61	*AtARF2*
*NsARF5*	*NISI06G1416.1*	Chr6	2742	913	5.57	*AtARF5*
*NsARF6*	*NISI10G0023.1*	Chr10	2664	887	6.12	*AtARF6*
*NsARF7a*	*NISI05G1550.1*	Chr5	2733	910	5.92	*AtARF7*
*NsARF7b*	*NISI05G0679.1*	Chr5	3153	1050	5.88	*AtARF7*
*NsARF8*	*NISI07G0010.1*	Chr7	2418	805	5.90	*AtARF8*
*NsARF9a*	*NISI11G0613.1*	Chr11	2046	681	6.39	*AtARF9*
*NsARF9b*	*NISI04G1762.1*	Chr4	1860	619	5.51	*AtARF9*
*NsARF16*	*NISI03G0644.1*	Chr3	2115	704	6.71	*AtARF10*

**Table 2:** Amino acid content of the *NsARF* gene family MR domain.

Gene	Gln (Q)	Pro (P)	Gly (G)	Leu (L)	Enrichment *	HMM
*NsARF1a*	0.06	0.12	0.05	0.08	P	DBD-MR-CTD
*NsARF1b*	0.05	0.08	0.05	0.09	L	DBD-MR-CTD
*NsARF2a*	0.06	0.06	0.06	0.08	L	DBD-MR
*NsARF2b*	0.04	0.10	0.07	0.06	P	DBD-MR-CTD
*NsARF5*	0.09	0.08	0.05	0.09	QL	DBD-MR-CTD
*NsARF6*	0.12	0.11	0.06	0.09	Q	DBD-MR-CTD
*NsARF7a*	0.10	0.09	0.06	0.08	Q	DBD-MR-CTD
*NsARF7b*	0.14	0.10	0.06	0.09	Q	DBD-MR-CTD
*NsARF8*	0.15	0.08	0.06	0.12	Q	DBD-MR-CTD
*NsARF9a*	0.04	0.12	0.03	0.05	P	DBD-MR-CTD
*NsARF9b*	0.05	0.07	0.04	0.05	P	DBD-MR-CTD
*NsARF16*	0.04	0.08	0.07	0.12	L	DBD-MR

* L, leucine; P, proline; Q, glutamine.

**Table 3:** *NsARF* promoter cis-element analysis.

Gene ID	Plant Hormones					Environmental Stress				MYB-Binding Site
	ABA	MeJA	Aux	GA	SA	Light	Defense	Circadian	Low Temperature	Drought Inducibility
*NsARF1a*	√	√				√	√			√
*NsARF1b*	√		√	√	√	√				√
*NsARF2a*	√	√				√		√		
*NsARF2b*	√					√				
*NsARF5*	√	√		√		√				
*NsARF6*	√	√		√	√	√			√	√
*NsARF7a*	√					√	√	√	√	√
*NsARF7b*		√			√	√	√		√	√
*NsARF8*	√	√	√	√		√	√		√	√
*NsARF9a*	√		√			√				
*NsARF9b*	√	√				√	√			√
*NsARF16*		√	√	√		√	√	√		

**Table 4:** qRT-PCR primers used to quantify *NsARF* gene expression.

Gene Name	qRT-PCR Primers	
*NsARF1a*	R	GGTTGCTCATCCCCTGTTCT
	F	TCATCAACAGATGGCACCCC→
*NsARF1b*	R	CAGAGACAAGTGGCCAGAGA
	F	GGAAGTGGAGCCAACTGTTG
*NsARF5*	R	GGGCGGTTGCATTGCATAAT
	F	AGCCGGTGAACTCTGAAAGG
*NsARF7a*	R	ATTACCGTGTCTCCTGCCAA
	F	CCAAACCGCTTACAGGGATG→
*NsARF7b*	R	GCCTTGGATGCTTGGATCTG
	F	AGGTTGGCTGGGATGAATCA
*NsARF9a*	R	AATCGGACCTATGCTCGGAG
	F	CCGACGATGAGGGTGATACA
*NsARF9b*	R	TCCCATGGATCATCGCCTAC
	F	GTTGGTCGGGCAGTTGATTT
*NsARF16*	R	GGATCCGCCGAGTAATCCAG
	F	TCCGGTGAGTAACAACGAGC

## Data Availability

All data generated or analysed during this study are included in this published article and its Supplementary Information Files.

## Supplementary Materials

The following supporting information can be downloaded at: https://www.mdpi.com/article/10.3390/ijms231911122/s1.

## References

[1] Abel S.P.G.E., Theologis A. (1996). Early genes and auxin action. Plant Physiol..

[2] Luo J., Zhou J., Zhang J. (2018). Aux/IAA Gene Family in Plants: Molecular Structure, Regulation, and Function. Int. J. Mol. Sci..

[3] Shiv B., Hagen G., Guilfoyle T. (2003). The Roles of Auxin Response Factor Domains in Auxin-Responsive Transcription. Plant Cell.

[4] Liscum E., Reed J.W. (2002). Genetics of Aux/IAA and ARF action in plant growth and development. Plant Mol. Biol..

[5] Woodward A.W. (2005). Auxin: Regulation, Action, and Interaction. Ann. Bot..

[6] Die J.V., Gil J., Millan T. (2018). Genome-wide identification of the auxin response factor gene family in *Cicer arietinum*. BMC Genom..

[7] Vernoux T., Brunoud G., Farcot E., Morin V., Van den Daele H., Legrand J., Oliva M., Das P., Larrieu A., Wells D. (2011). The auxin signalling network translates dynamic input into robust patterning at the shoot apex. Mol. Syst. Biol..

[8] Ulmasov T., Hagen G., Guilfoyle T.J. (1999). Activation and Repression of Transcription by Auxin-Response Factors. Proc. Natl. Acad. Sci. USA.

[9] Finet C., Berne-Dedieu A., Scutt C.P., Marlétaz F. (2013). Evolution of the ARF Gene Family in Land Plants: Old Domains, New Tricks. Mol. Biol. Evol..

[10] Weijers D., Benkova E., Jager K.E., Schlereth A., Hamann T., Kientz M., Wilmoth J.C., Reed J.W., Jurgens G. (2005). Developmental specificity of auxin response by pairs of ARF and Aux/IAA transcriptional regulators. EMBO J..

[11] De Smet I., Lau S., Voß U., Vanneste S., Benjamins R., Rademacher E.H., Schlereth A., De Rybel B., Vassileva V., Grunewald W. (2010). Bimodular auxin response controls organogenesis in *Arabidopsis*. Proc. Natl. Acad. Sci. USA.

[12] Fukaki H., Taniguchi N., Tasaka M. (2006). PICKLE is required for SOLITARY-ROOT/IAA14-mediated repression of ARF7 and ARF19 activity during *Arabidopsis* lateral root initiation. Plant J..

[13] Liu X., Zhang H., Zhao Y., Feng Z., Li Q., Yang H., Luan S., Li J., He Z. (2013). Auxin controls seed dormancy through stimulation of abscisic acid signaling by inducing ARF-mediatedABI3 activation in *Arabidopsis*. Proc. Natl. Acad. Sci. USA.

[14] Wang L., Hua D., He J., Duan Y., Chen Z., Hong X., Gong Z. (2011). Auxin Response Factor2 (ARF2) and its regulated homeodomain gene HB33 mediate abscisic acid response in *Arabidopsis*. PLoS Genet..

[15] Wang D., Pei K., Fu Y., Sun Z., Li S., Liu H., Tang K., Han B., Tao Y. (2007). Genome-wide analysis of the auxin response factors (ARF) gene family in rice (*Oryza sativa*). Gene.

[16] Peng Y., Fang T., Zhang Y., Zhang M., Zeng L. (2020). Genome-Wide Identification and Expression Analysis of Auxin Response Factor (ARF) Gene Family in Longan (*Dimocarpus longan* L.). Plants.

[17] Liu Y., Jiang H., Chen W., Qian Y., Ma Q., Cheng B., Zhu S. (2011). Genome-wide analysis of the auxin response factor (ARF) gene family in maize (*Zea mays*). Plant Growth Regul..

[18] Tuteja N. (2007). Abscisic Acid and Abiotic Stress Signaling. Plant Signal. Behav..

[19] Travaglia C., Cohen A.C., Reinoso H., Castillo C., Bottini R. (2007). Exogenous Abscisic Acid Increases Carbohydrate Accumulation and Redistribution to the Grains in Wheat Grown Under Field Conditions of Soil Water Restriction. J. Plant Growth Regul..

[20] Kizis D., Pagès M. (2002). Maize DRE-binding proteins DBF1 and DBF2 are involved in rab17 regulation through the drought-responsive element in an ABA-dependent pathway. Plant J. Cell Mol. Biol..

[21] Battal P., Erez M.E., Turker M., Berber I. (2008). Molecular and Physiological Changes in Maize (*Zea mays*) Induced by Exogenous NAA, ABA and MeJa during Cold Stress. Ann. Bot. Fenn..

[22] Luo X., Bai X., Sun X., Zhu D., Liu B., Ji W., Cai H., Cao L., Wu J., Hu M. (2013). Expression of wild soybean WRKY20 in *Arabidopsis* enhances drought tolerance and regulates ABA signalling. J. Exp. Bot..

[23] Garcia-Moreno B. (2009). Adaptations of proteins to cellular and subcellular pH. J. Biol..

[24] Tang Y., Bao X., Liu K., Wang J., Zhang J., Feng Y., Wang Y., Lin L., Feng J., Li C. (2018). Genome-wide identification and expression profiling of the auxin response factor (ARF) gene family in physic nut. PLoS ONE.

[25] Jain M., Khurana J.P. (2009). Transcript profiling reveals diverse roles of auxin-responsive genes during reproductive development and abiotic stress in rice. FEBS J..

[26] Li S., Xie Z., Hu C., Zhang J. (2016). A Review of Auxin Response Factors (ARFs) in Plants. Front. Plant Sci..

[27] Chapman E.J., Estelle M. (2009). Mechanism of Auxin-Regulated Gene Expression in Plants. Annu. Rev. Genet..

[28] Bargmann B.O.R., Vanneste S., Krouk G., Nawy T., Efroni I., Shani E., Choe G., Friml J., Bergmann D.C., Estelle M. (2013). A map of cell type-specific auxin responses. Mol. Syst. Biol..

[29] Ding T., Zhang F., Wang J., Wang F., Liu J., Xie C., Hu Y., Shani E., Kong X., Ding Z. (2021). Cell-type action specificity of auxin on *Arabidopsis* root growth. Plant J..

[30] Procko C., Burko Y., Jaillais Y., Ljung K., Long J.A., Chory J. (2016). The epidermis coordinates auxin-induced stem growth in response to shade. Genes Dev..

[31] Swarup R., Kramer E.M., Perry P., Knox K., Leyser H.M.O., Haseloff J., Beemster G.T.S., Bhalerao R., Bennett M.J. (2005). Root gravitropism requires lateral root cap and epidermal cells for transport and response to a mobile auxin signal. Nat. Cell Biol..

[32] Daszkowska-Golec A. (2016). The Role of Abscisic Acid in Drought Stress: How ABA Helps Plants to Cope with Drought Stress.

[33] Yoshioka T.T.U.S., Endo T., Satoh S. (1998). Restoration of seed germination at supraoptimal temperatures by fluridone, an inhibitor of abscisic acid biosynthesis. Plant Cell Physiol..

[34] Muthamilarasan M., Mangu V.R., Zandkarimi H., Prasad M., Baisakh N. (2016). Structure, organization and evolution of ADP-ribosylation factors in rice and foxtail millet and their expression in rice. Sci. Rep..

[35] Gamble P.E., Mullet J.E. (1986). Inhibition of carotenoid accumulation and abscisic acid biosynthesis in fluridone-treated dark-grown barley. Eur. J. Biochem..

[36] Chen C., Chen H., Zhang Y., Thomas H.R., Frank M.H., He Y., Xia R. (2020). TBtools: An Integrative Toolkit Developed for Interactive Analyses of Big Biological Data. Mol. Plant.

