# Supplementary figures and images for: The Identification and Expression Analysis of the Nitraria sibirica Pall. Auxin-Response Factor (ARF) Gene Family
Source: Int J Mol Sci. 2022 Sep 22;23(19):11122. doi: 10.3390/ijms231911122 (PMC9570472; doi:10.3390/ijms231911122)

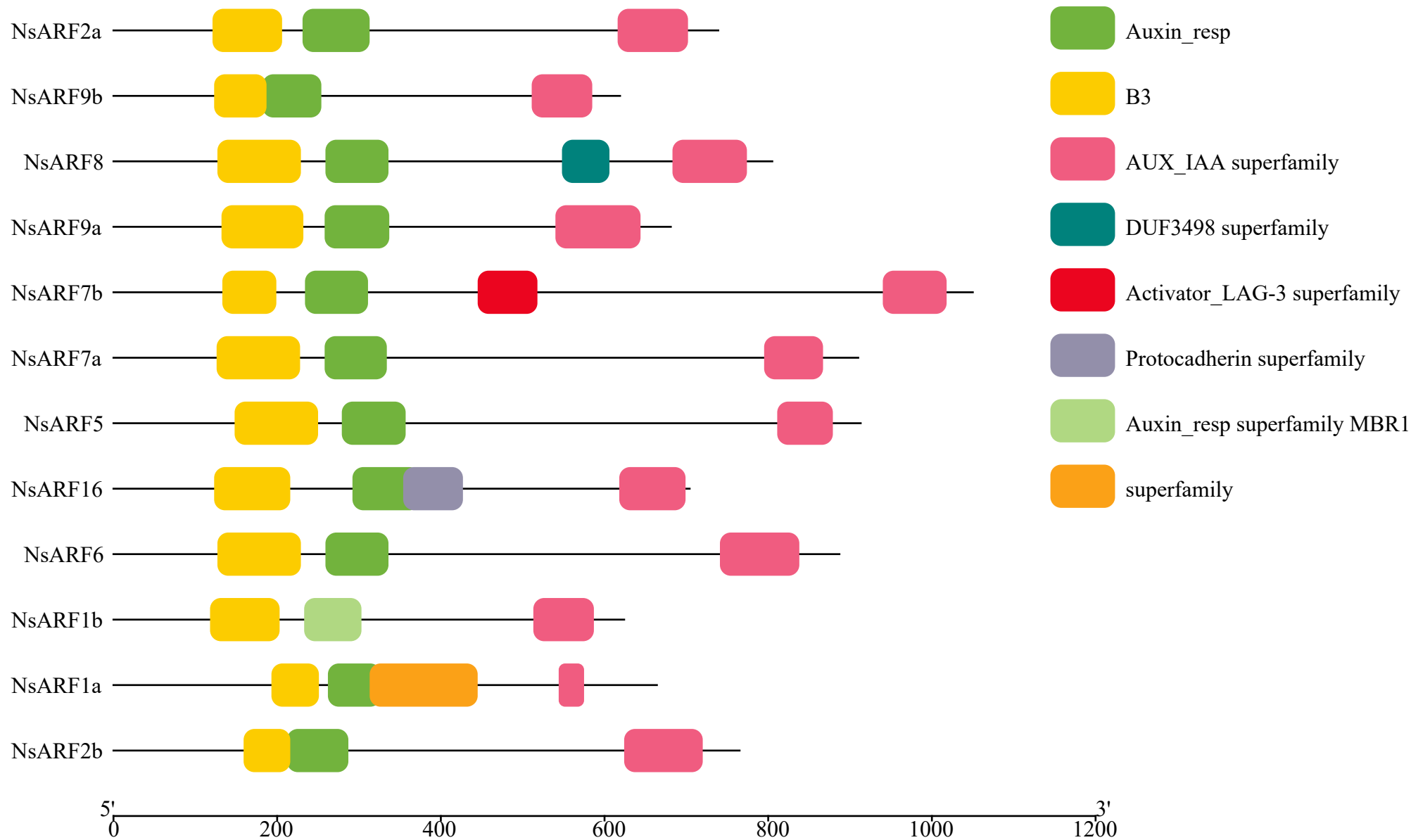

**Figure S1.** Protein structure of *NsARF*

Supplement: Supplementary file 1 [file ijms-23-11122-s001.zip › ijms-1869934-supplementary.pdf]
